# Peripheral clock gene oscillations are perturbed in neonatal and adult rat offspring raised under adverse limited bedding conditions

**DOI:** 10.1038/s41598-023-47968-y

**Published:** 2023-12-21

**Authors:** Claire-Dominique Walker, Tara C. Delorme, Silke Kiessling, Hong Long, Nicolas Cermakian

**Affiliations:** 1 Douglas Mental Health University Institute, 6875 Lasalle Blvd, Montreal, QC H4H 1R3 Canada; 2 Dept of Psychiatry, McGill University, Montreal, QC Canada; 3 Faculty of Health and Medical Sciences, University of Surrey, Stag Hill Campus, Guildford, GU27XH UK

**Keywords:** Neuroscience, Circadian rhythms and sleep, Circadian regulation

## Abstract

Circadian (24-h) rhythms in the suprachiasmatic nucleus (SCN) are established in utero in rodents, but rhythmicity of peripheral circadian clocks appears later in postnatal development. Since peripheral oscillators can be influenced by maternal feeding and behavior, we investigated whether exposure to the adverse environmental conditions of limited bedding (LB) during postnatal life would alter rhythmicity in the SCN, adrenal gland and liver in neonatal (postnatal day PND10), juvenile (PND28) and adult rats. We also examined locomotor activity in adults. Limited bedding increased nursing time and slightly increased fragmentation of maternal behavior. Exposure to LB reduced the amplitude of *Per2* in the SCN on PND10. Adrenal clock gene expression (*Bmal1*, *Per2, Cry1, Rev-erbα, Dbp)* and corticosterone secretion were rhythmic at all ages in NB offspring, whereas rhythmicity of *Bmal1, Cry1* and corticosterone was abolished in neonatal LB pups. Circadian gene expression in the adrenal and liver was well established by PND28. In adults, liver expression of several circadian genes was increased at specific daytimes by LB and the microstructure of locomotor behavior was altered. Thus, changes in maternal care and behavior might provide important signals to the maturing peripheral oscillators and modify, in particular their output functions in the long-term.

## Introduction

Circadian rhythms are established early in developing rodents and regulate important physiological functions such as sleep–wake cycles, body temperature, hormone secretion, metabolic regulation and behaviors^1^. Circadian oscillations of clock genes such as *Per1, Per2, Clock, Bmal1* and *Rev*-*Erbα* as well as many clock-controlled genes are central to the establishment and maintenance of homeostatic mechanisms throughout the brain and peripheral organs^2,3^. The “master clock” in the suprachiasmatic nucleus (SCN) coordinates the rhythms of oscillators in other brain regions^4^ and in peripheral tissues, like the liver and adrenal glands^5,6^. In peripheral tissues however, other environmental cues, such as temperature or the timing and composition of food can have a robust influence on tissue oscillators^7^. In particular, changes in meal times in adults can alter the phase relationship of circadian rhythms within the central and peripheral clocks^8^.

Some circadian rhythms emerge in rats and other mammals in utero. Rats begin to show rhythmicity in the patterns of glucose utilization in the SCN as early as gestational day 19^9^ and these rhythms are entrained primarily by the mother’s circadian clock^10–13^. Interestingly, fetuses in utero entrain to the maternal SCN clock under normal conditions^14^, but also to maternal behavior/feeding regime^15^, in particular in conditions where the influence of the SCN is reduced or absent^15–17^. In natural environmental conditions, food/behavior-related signals may be more significant than the SCN-driven signal to adapt to uncertain metabolic conditions^18^. In particular, both fetal SCN and liver show patterns of gene expression that are in line with the mother’s feeding behaviour^19^, underscoring the importance of maternal behaviour on early circadian development.

Postnatally, pups begin to show the first signs of photic entrainment as early as postnatal day (PND) 6^10^, but even after this, pups still rely on maternal cues for circadian entrainment until about PND15 and they become fully reliant on light entrainment around 20 days of life, when they transition into nocturnal sleep/wake patterns^20^. This dependency upon maternal cues for the development of circadian activity during the first 2 weeks of life suggests that alterations in maternal behavior and patterns of care towards the pups might have important consequences for the pups during this sensitive period of development. Blinded pups or pups cross fostered to a mother with a different circadian rhythm than their biological mother entrain to the nursing patterns of the foster mother^21^. They maintain these circadian patterns, even after they have become more independent by weaning age, suggesting that the maternal entrainment is maintained in pups even after the onset of independent feeding.

It is well established that maternal care in rodents follows a circadian pattern and occurs in a specific sequence (retrieval, pup licking, arched back nursing, induction of REM sleep activity and pup suckling leading to the milk ejection reflex)^22^. However, maternal care patterns can be altered by exposure to early chronic stressors, such as restricted feeding^23^, repeated daily 3 h separation from the pups^24^ and reduced availability of nesting and bedding material during the first 10 days postpartum^25^ or other periods postnatally^26,27^. Exposure to limited bedding (LB) during the first 10 days of life has been shown to result in unpredictable patterns of care towards the pups^25^. This experimental paradigm has been designed to closely approximate the situation of child neglect in humans, when infant care is fragmented and unpredictable^28^. Exposure to these early life stressors has been linked to many negative outcomes in the adult offspring, including increased anxiety^29^, and impairments in cognition and social development^30^. In humans, most studies investigating the effects of childhood adversity on circadian rhythms have reported changes in cortisol secretion, whether a blunting of the circadian rhythm or exacerbation of cortisol awakening responses in adults^31^. Very few studies, if any, have examined early consequences on circadian physiology in infants or toddlers. The availability of animal models mimicking some of the components of early adversity in humans allows investigating the consequences of disruptions of maternal care on the establishment of circadian rhythmicity in developing pups.

The goal of this study is to explore whether early life adversity using the LB paradigm affects the establishment of peripheral clocks and their function in the offspring and whether some of the changes in clock genes are maintained in the long term, affecting locomotor behavior. We hypothesize that the limited availability of resources during the first 10 days of life will affect circadian rhythmicity in the neonatal offspring either through changes in maternal behavioral cues or changes in metabolic conditions (availability of nursing and/or altered thermoregulation). We report altered or abolished rhythms in clock gene expression in the liver and adrenal glands of LB neonates on PND10, but robust circadian rhythms in juveniles and changes in gene expression in adults. Some of the gene expression changes in LB offspring were maintained until adulthood together with altered adult locomotor behavior.

## Methods

### Animals

Pregnant Sprague–Dawley female rats (Charles River, St-Constant, QC, Canada) were received on gestation day 14–15 and maintained under controlled conditions of light (05:00 h lights on, 17:00 h lights off to match the light cycle of the supplier), temperature (22–24 °C), and humidity (70–80%). Pregnant dams were provided with rat chow and water ad libitum and left undisturbed until parturition (PND0). Litters were culled to 10 pups on PND1. For measures taken at PND10, a total of 14 mothers was used divided into 2 cohorts (cohort 1 = 6 mothers and cohort 2 = 8 mothers). Animals in cohort 1 were used for in situ hybridization studies while animals in cohort 2 (45 males and 20 females) were used for determination of clock gene expression and corticosterone assay. For measures taken at PND28 and in adulthood, a total of 12 mothers was used and weaned offspring (43 males and 49 females) were kept for determination of clock gene expression, corticosterone levels (P28 and adulthood) and locomotor behavior up to adulthood. Two cohorts of adult male rats were examined for locomotor running wheel activity (cohort 1 = 12 rats, cohort 2 = 16 rats). On PND10 and 28, results from both male and female offspring were pooled and only male offspring were used for the adult determinations. All procedures were approved by the University Animal Care Committee at McGill University in accordance with the guidelines of the Canadian Council on Animal Care. The study is reported in accordance with the ARRIVE guidelines and conforms to its principles.

### Limited bedding paradigm and maternal behaviour

The LB paradigm was used between PND1–10 according to a protocol adapted from Baram et al.^32^ with cage changes on PND4 and PND7. On PND1, mothers and their litters were randomly assigned to the limited bedding (LB, n = 7) or normal bedding (NB, n = 7) condition. LB mothers and their litters were placed on an aluminum mesh platform 2.5 cm above the cage floor. Approximately 1.5 cm of bedding was added below the platform to cover the cage floor. The dams were given one-half of one paper towel (12 cm × 20 cm) as nesting material. The NB cages received a 2.5 cm layer of woodchips and one-half of a paper towel. All litters were kept with their biological mother for the duration of the experiments. The weights of all pups and dams were recorded on PND1, 4, and 7 and animals were otherwise left undisturbed until tissue collection on PND10 or weaning on PND21. After weaning, rats were group-housed according to treatment and sex.

Maternal behavior was observed in mothers from cohort 2 (4/bedding condition) between PND5-6 using continuous infrared video recordings. Specific parameters of maternal behavior such as active/passive nursing, pup grooming, pup retrieving, self-grooming, sleeping, eating, drinking, wandering, were scored every minute for five segments of 1 h for each light and dark phase. Behavioral observation segments coincided with tissue collection times (Circadian time, CT2, 8, 14, 20) and additional observations were performed around the earlier (CT2) and later (CT20) time points. The fragmentation of overall behavior, i.e., the degree to which maternal behavior occurs in many short bouts^33^ during each observation period was determined using a behavioral consistency score in which a score of “1” was given when behavior changed from one minute to the next, and “0” when there was no change in the type of behavior exhibited^25^.

### Circadian locomotor behavior

Rats were individually housed in running wheel cages on PND35 and were left to acclimatize one week without locomotor recording, after which, recordings in the normal 12:12LD cycle started for 2 weeks (14 days). Light ON intensity was 232.6–240.5 Lux (Photometer, Internat. Light Inc, Model IL1400A) and light OFF intensity measured was 0.05 Lux. In order to determine whether adult rat offspring from the different bedding groups would display altered behavioral locomotion rhythms and sensitivity to a light challenge (either constant light or 6 h phase advance), rats were exposed to 3 lighting conditions, each for 2 weeks: 12:12LD with a phase advance of 6 h, (starting on PND56), constant darkness (DD, 0.05Lux, starting on PND70) and constant light (LL, 232.6–240.5 Lux, starting on PND84). While 12:12LD is a condition where external timing cues guide entrainment, exposure to DD allows for the assessment of free-running rhythms without the influence of light as a timing cue. Exposure to LL or to a 6 h-phase advance challenge has been shown to weaken the suprachiasmatic nucleus neuronal network^34^ and as such, may uncover network connectivity impairments in LB offspring.

Cage changes were done at the end of each 2 week-period, a few hours before lighting regimen was changed. We ran 2 cohorts of animals with n = 6/group and n = 8/group in the first and second cohort, respectively.

Running wheel activity data was collected and analyzed using ClockLab software, version 6 (Actimetrics, Wilmette, IL, USA). The analysis was carried out on the last 10 days of wheel-running recordings for each condition. We calculated various circadian locomotor activity variables, including circadian period (tau; calculated using a chi-square periodogram), duration of the active period (*alpha*; denoted by the numbers of hours between activity onset and offset), total amount of daily activity and percent day activity relative to total daily activity. In constant conditions (DD and LL), the subjective night was defined to be from the beginning of activity onset until half a period later, and the rest of the circadian cycle was defined as the subjective day. Analyses for non-parametric variables included inter-daily stability, which quantifies the synchronization to the 24 h light–dark cycle, intra-daily variability, which quantifies rhythm fragmentation, and relative amplitude, which quantifies the robustness of the rhythm. A detailed analysis of activity bouts was also done, where a bout is defined as a sustained period of activity. Namely, we calculated the number of counts per bout of activity, the duration of activity bouts, and the number of activity bouts per day.

### Tissue collection

Lighting conditions were changed to constant darkness (no light phase) 48 h prior to the start of dissections. Animals were decapitated on PND10, PND28 and PND98-99 (adult) at circadian time (CT) CT2, CT8, CT14, CT20, where circadian time refers to the time under constant conditions (DD) corresponding to 2, 8, 14 and 20 h after lights on in the previous LD cycle. Sample size were: PND10: n = 8/CT/rearing condition; PND28 n = 11–12/CT/rearing condition; PND98-99 n = 4–5/CT/rearing condition. No more than 2 pups per litter was used for each time point. Tissue collection in adult rats was performed after locomotor behavior was terminated on PND98-99 and under the same experimental conditions as described above. Trunk blood was collected on EDTA (60 mg/ml, 20 µl/ml blood) and stored on ice, then spun down and the plasma was stored at − 20 °C until analyzed for corticosterone concentration. Thymus and adrenals were collected in pre-weighted empty tubes and weight of the organs was recorded. Brain, adrenal glands and liver were removed and immediately frozen (on isopentane for brain collection) on dry ice, then stored at − 80 °C until analysis.

### Plasma corticosterone determination

Plasma corticosterone concentration was measured using a specific radioimmunoassay (RIA) for corticosterone (MP Biomedicals, Solon, OH, USA, CAT #07-120102) as previously described^35^. Five μl of samples were assayed in duplicates and the limit of detection of the assay was 0.31 μg/dl. All age-related samples were run within the same assay.

### Real time PCR analysis of liver and adrenal clock gene expression

RNA was extracted from the liver and adrenal glands using Trizol (Life Technologies) according to the manufacturer’s protocol and using either 400 μl (liver) or 100  μl (adrenal) of Trizol. The tissue was homogenized on ice using a handheld homogenizer (Cole-Parmer and Cat# is RK-04727-13). cDNA was synthesized using the MultiScribe reverse-transcription kit (Life Technologies) according to manufacturer’s instructions. Briefly, 10 × RT Buffer, 25 × dNTP Mix (100 mM), 10 × RT Random Primers, Multiscribe Reverse Transcriptase, RNase Inhibitor, and Nuclease Free water were combined into a master mix and then added to samples on ice where the contents of each tube were 1:1 sample: master mix. Samples were placed into a ThermoCycler (Applied Biosystems 9700) under the following conditions: Step 1 at 25 °C for 10 min, Step 2 at 37 °C for 120 min, Step 3 at 85 °C for 5 s, and then infinite hold at 4 °C. cDNA samples were removed from the ThermoCycler and diluted by 1:5 and then stored in the − 20 °C freezer until used for qPCR. Clock gene expression was assessed using SYBR Green quantitative PCR (qPCR) (Life Technologies, 7500 Real-Time PCR System). Primers for clock genes *Bmal1* (0.4 μM), *Cry1* (0.5 μM), *Dbp* (0.8 μM), *Rev-erbα* (0.625 μM) and *Per2* (0.8 μM), as well as liver metabolic genes *Ucp2 (0.4 uM) and Por* (0.2 uM) were used (sequences in Table 1). qPCR values were normalized with *Gapdh (glyceraldehyde-3-phosphate)*. Dilution series (1:2, 1:10, 1:50, 1:250, 1:1250) standard curves were performed in triplicate for each primer pair using reverse transcription cDNA products from rat liver tissue as well as rat adrenal tissue. The concentration of primers used was determined based on the efficiency values calculated from the line of best fit of the standard curve (efficiency ≥ 96%). The ratio of sample: primer: SYBR was 1:1:2 and qPCR was done in triplicate for each sample assayed. Relative expression levels were calculated using the 2^−delta delta Ct^ method^36^.

**Table 1:** Primer sequences used in the SYBR Green quantitative PCR assays.

Gene	Forward primer	Reverse primer
Clock genes		
* Per2*	5′-CACCCTGAAAAGAAAGTGCGA-3′	5′-CAACGCCAAGGAGCTCAAGT-3′
*Bmal1*	5′-CAATGCGATGTCCCGAAGTTAGA-3′	5′-TCCCTCGGTCACATCCCTGAGAAT-3′
Rev-erb*α*	5′-ACAGCTGACACCACCCAGATC-3′	5′-CATGGGCATAGGTGAAGATTTCT-3′
*Dbp*	5′-AGGCAAGGAAAGTCCAGGTG-3′	5′-TCTTGCGTCTCTCGACCTCTT-3′
*Cry1*	5′-CCTTCTAATCCTAATGGGAACG-3′	5′-ACCACTTCCTTGAGAGCAGTTT-3′
*Ucp2*	5′-ACTCCTGTGTTCTCCTGT-3′	5′-AATCGTCAAGACGAGACAGAGG-3′
*Por*	5′-CAGGACTTCTATGACTGGCTG-3′	5′-AAAGAACTCGCACACAGC-3′
Housekeeping gene		
*Gapdh*	5′-TGCCAAGTATGATGACATCAAGAAG-3′	5′-AGCCCAGGATGCCCTTTAGT-3′

### In situ hybridization of *Bmal1* and *Per2* in the neonatal brain

Frozen brains from PND10 pups were sliced into 18-μm coronal sections and collected directly onto charged slides (Fisherbrand™ Superfrost™ Plus Microscope Slides). In situ hybridization of *Bmal1* and *Per2*transcripts in the SCN was carried out using ^35^S-labeled cRNA probes. One series of slides underwent Cresyl violet staining for adequate localization of the targeted structures. All solutions were treated with diethyl pyrocarbonate (DEPC) and sterilized to prevent RNA degradation. Slide-mounted sections were desiccated in a vacuum at 4 °C overnight after sectioning, then stored at − 80 °C until the start of the hybridization protocol.

Sections were brought to room temperature and fixed in 4% paraformaldehyde made in phosphate buffered saline (PBS) (pH 7.4) for 20 min, then washed with 2X standard saline citrate (SSC) for 5 min, then 0.1 M triethanolamine (pH 8.0) for 5 min. Sections were incubated with a solution of 0.1 M triethanolamine (pH 8.0) and 0.25% acetic anhydride for 10 min, and then dipped 10 times in 2XSSC. Sections were then dehydrated through graded concentrations of alcohol made with DEPC water (70, 80, 95, and two sets of 100%). Sections were allowed to dry at room temperature for at least 1 h before the hybridization procedure.

After drying, 90 μl of hybridization mixture was spotted on each slide, sealed under a coverslip, and sections were incubated at 55 °C overnight (~ 15–20 h) in an oven. The hybridization mixture contained 800 µl of hybridization solution (12.5 ml of 50% deionized formamide, 2.5 ml 20 × SSC, 0.25 ml of 100 × Denhart’s solution, 5 ml of 10% of dextran sulphate, 1 ml sheared single-stranded DNA (40 mg/ml), 50 µl of tRNA (10 mg/ml), 50 µl of DTT 1 M) and 100 μl of *Per2* probe (10 × 10^6^ cpm/ml) or *Bmal1* probe (10 × 10^6^ cpm/ml) and 100 µl DEPC water. Plasmids for probe preparation were generously provided by Dr H. de la Iglesia, U. of Washington (Per2) and Dr H. Oster, U. of Lubeck (Bmal1). After incubation, coverslips were removed, and the slides were rinsed four times in 1XSSC at room temperature. After this, slides were bathed in a solution composed of 50% formamide and 50% 2xSSC for 20 min at 52 °C, then again for 20 min in 1X SSC at 52 °C. Sections were washed in 2X SSC twice for one minute each and slides were then placed in 250 ml of RNaseA solution (500 μl of 10 mg/ml) in RNAse buffer (100 ml 5 M NaCl, 10 ml of 1 M Tris–HCl (pH 8.0), and 2 ml of 0.5 M EDTA (pH 8.0)) at 37 °C for 30 min. Slides were then washed twice for 5 min each in 2XSSC, and then for 5 min in formamide wash (50% 2XSSC and 50% formamide) at 52 °C. Slides were then dehydrated in increasing concentrations of ethanol diluted with 0.1xSSC for 3 min per concentration (70%, 80%, 95%), then submerged in distilled water for 15 s. They were then submerged in 70% ethanol for 3 min, followed by 15 s dips in a 95% ethanol bath then two 100% ethanol baths before being left to dry at room temperature for at least 2 h.

Once sections were dry, they were exposed to Kodak X-ray film (Eastman Kodak Co, Rochester, NY) for a period varying between 4 and 7 days together with radioactive ^14^C standards. Matching sections selected for mostly the middle-caudal portion of the SCN were analyzed using a MCID Image analysis system (Imaging Research Inc, St-Catherine, Canada). Blind quantitative analysis of hybridization signal was performed for at least 3–4 sections/animal.

### Statistical analysis

All results were analyzed by two-way ANOVAs. Post-hoc analyses were conducted using the Bonferroni test for multiple comparisons. Non-linear fit analyses were performed using Graphpad Prism version 5 (GraphPad Prism Software Inc., San Diego, CA). Cosine regression analysis was done by using Daniel Soper’s Calculator for F- value and P-value for multiple regression V.4 (Copyright © 2006–2020 by Dr. Daniel Soper). Body weight, thymus and adrenal weight data were analyzed for each age separately using a two tail t-test. The level of significance is set at *p* < 0.05 in all analyses.

## Results

### Effect of bedding conditions on maternal behavior and pup weight

**Figure 1:**
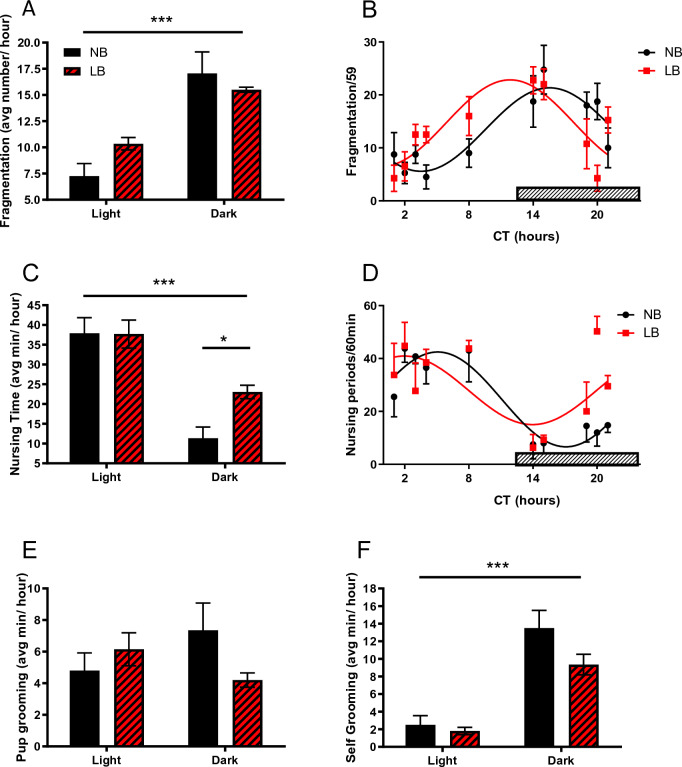
Maternal behavior in NB (n = 4) and LB (n = 4) litters on postpartum days 5–6. One hour observation periods over the light dark cycle (5 observations/light phase) were scored every minute for nursing time (**C**,**D**), pup grooming (**E**) and self-grooming time (**F**) and each of these parameters is expressed as average min per hour. Fragmentation score (**A**,**B**) was compiled by giving a score of zero if no change was observed and a score of 1 when behavior changed from one minute to the next. Thus, the maximum fragmentation score for a 60 min observation is 59. Fragmentation, total nursing time and self-grooming time were significantly different across the light cycle. Values represent the mean +/− SEM of 5 one hour observations per light phase (**A**,**C**,**E**,**F**) or the mean +/− SEM of each observation period over the entire light:dark cycle (**B**,**D**). *p < 0.05; ***p < 0.001.

Analysis of maternal behavior on PND5-6 (4 litters/bedding condition in cohort 2) was divided into four main categories: nursing (both active and passive), pup grooming, self-grooming of the mother, and fragmentation of care towards pups (Fig. 1). Overall, mothers in both groups displayed higher levels of fragmentation during the dark phase than in the light, with no significant bedding effect (Fig. 1A) (Light effect: F(1, 12) = 46.43, *p* < 0.001; Bedding effect: F(1, 12) = 0.001, *p* = 0.983). Fragmentation in both groups was rhythmic (*p* < 0.001) over the 10 time points analyzed (Fig. 1B) and there was a significant phase shift (*p* = 0.0018) with LB mothers displaying earlier peak of behavioral fragmentation (prior to lights off) compared to NB mothers (Cosine regression analysis: NB: baseline = 13.46, amplitude = 7.88, phase shift = 15.53 h; LB: baseline = 14.68, amplitude = 8.16, phase shift = 11.84 h). On PND5-6 NB and LB mothers spent significantly more time nursing during the light phase than in the dark (Fig. 1C) (Light effect: F (1, 12) = 43.23, *p* < 0.001). There was no main effect of bedding (F (1, 12) = 3.368, *p* = 0.091), however, in the dark, NB mothers tended to nurse less than LB mothers, in agreement with previous findings^29,33^. Nursing was rhythmic in both groups (*p* < 0.05) (Fig. 1D) and a significant phase shift was also observed between LB and NB mothers (*p* = 0.0433). Cosine regression analysis parameters for nursing were: NB: baseline = 24.55, amplitude = 17.92, phase shift = 5.09 h; LB: baseline = 27.98, amplitude = 12.95, phase shift = 1.96 h. There was no difference in the amount that mothers in either condition groomed their pups (Fig. 1E), either in the light or in the dark (Bedding: F (1, 12) = 0.524, *p* = 0.4828; Light: F (1, 12) = 1.844, *p* = 0.2). This is consistent with findings from our lab as well as others^28,30^. Both NB and LB mothers groomed themselves more in the dark (F (1, 12) = 55.05 *p* < 0.0001) (Fig. 1F) although there was no significant effect of bedding (F (1, 12) = 3.296, *p* = 0.095).

There was no significant bedding group differences in pup weight at PND9, one day prior to tissue collection, although pup weight gain (weight on PND9 minus weight on PND1) was significantly reduced in LB pups (NB = 17.65 ± 0.58 g; LB = 14.17 ± 1.07 g; *p* = 0.014). Thymus weight (NB = 3.47 +/− 0.14 mg/g BW; LB = 3.27 +/− 0.08) and adrenal weight (NB = 0.35 +/− 0.013; LB = 0.326 +/− 0.01 mg/g BW) on PND10 pups were not significantly altered by bedding conditions. In PND28, absolute body weight differences between bedding groups remained significant for both males (NB = 104.3 +/− 2.21 g; LB = 91.3 ± 2.81; *p* < 0.001) and females (NB = 95.5 +/− 2.62; LB = 83.8 +/− 1.98, *p* < 0.001), but there was no significant effect of bedding on thymus weight or adrenal weight in either males of female juvenile offspring. Adult LB male offspring were also significantly smaller than their NB counterparts (NB = 626.9 +/− 11 g, LB = 588 +/− 10.2 g; F(1,26) = 6.61, *p* = 0.016), but without significant differences in thymus or adrenal weights. In summary, mothers from both groups displayed circadian rhythmicity in maternal behavior and although the effect of bedding on nursing and fragmentation was modest, there was a significant shift is these behaviors with bedding condition. Pup weight gain before weaning and body weight in juvenile and adult rats remained significantly reduced by the LB procedure.

### Circadian *Bmal1* and *Per2* expression in the SCN on PND10

**Figure 2:**
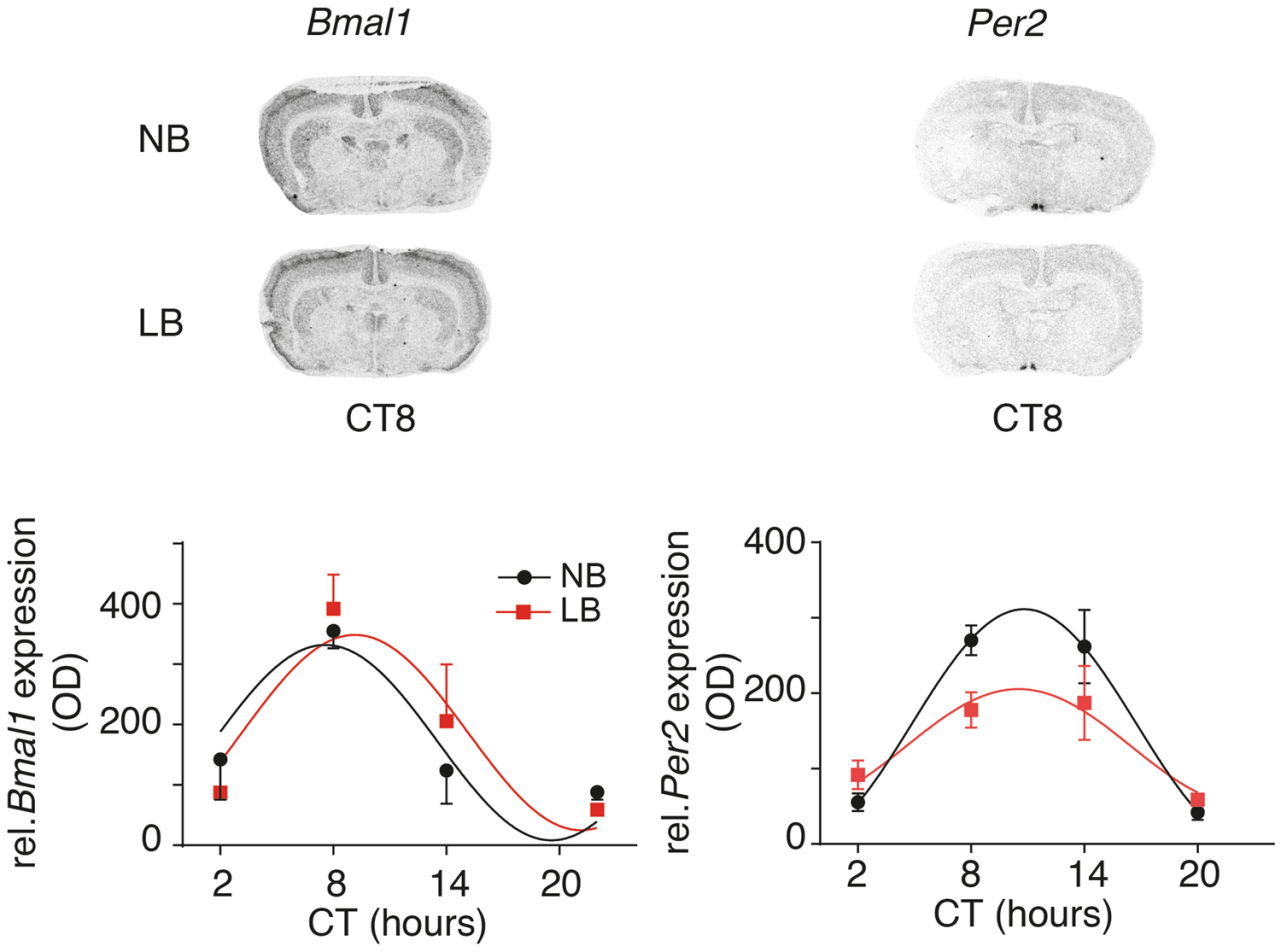
Quantification of *BMal1* and *Per2* gene expression in the SCN of PND10 NB or LB pups by in situ hybridization. Top: Photomicrographs of autoradiograms displaying the hybridized signal for *BMal1* (left) and *Per2* (right) in NB (top) or LB (bottom) pups at and the maximum expression on CT8. Bottom: Quantification of the hybridized signal as a function of CT by optical densitometry. Groups found to display a rhythm according to cosine regression analysis (*p* < 0.05) are represented with curved lines. Blind quantitative analysis of the hybridization signal was performed for at least 3–4 sections/animal. CT, circadian time. Values represent the mean +/− SEM of 3–6 animals/time point with pooled results from male and female pups.

As we observed a phase advance in nursing behavior of the LB mothers compared to the NB mothers, we examined whether these changes resulted in alterations in *Bmal1* and *Per2* gene expression in the SCN of the offspring. In situ hybridization in the SCN was conducted on 2 cohorts of PND10 rats. As depicted in Fig. 2, significant rhythms of *Bmal1* were observed in both NB (R^2^ = 0.618, F = 4.31, *p* = 0.0436) and LB (R^2^ = 0.668, F = 5.35, *p* = 0.0257) pups, with no amplitude or phase shift differences. A two-way ANOVA with time and bedding as between subject factors showed a significant effect of time (F(3, 14) = 11.77, *p* < 0.001), but no main effect of bedding (F(1, 14) = 0.05, *p* = 0.825) and no interaction between time and bedding (F(3, 14) = 0.662, *p* = 0.588). The highest level for *Bmal1* expression was observed close to CT8 for both groups. For *Per2* expression in the SCN, cosine regression analysis showed a significant rhythm in both NB (F = 14.01, *p* < 0.001, R^2^ = 0.737) and LB (F = 5.16 *p* < 0.01, R^2^ = 0.463) reared pups. However the amplitude of *Per2* expression was significantly suppressed in LB compared to NB pups (*p* = 0.0127). A two-way ANOVA with time and bedding as between subject factors showed a significant effect of time (F(3, 33) = 19.63, *p* < 0.0001), but no main effect of bedding (F(1, 33) = 1.763, *p* = 0.193). There was no significant interaction between time and bedding (F(3, 33) = 2.21, *p* = 0.106).

### Effect of bedding conditions on rhythmicity of clock and clock-controlled genes in the adrenal gland in neonates, juveniles and adult rats

**Figure 3:**
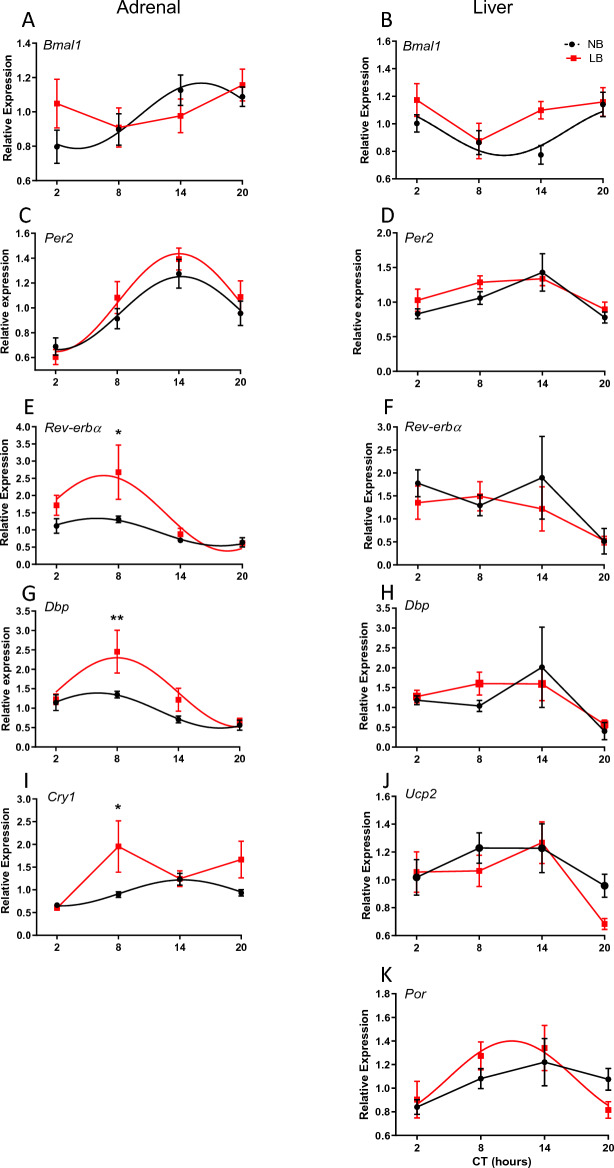
Gene expression of clock and clock-associated genes in the adrenal gland (left, **A**,**C**,**E**,**G**,**I**) and liver (right, **B**,**D**,**F**,**H**,**J**,**K**) of PND10 pups raised under NB or LB conditions. Black lines represent data for NB pups while red lines represent data for LB pups. Groups found to display a rhythm according to cosine regression analysis (*p* < 0.05) are represented with curved lines, while those which are not rhythmic are represented by straight lines going directly through the plotted points. For all genes, n = 6–8 samples/time point with pooled results from both male and female pups. Statistical significance and cosine regression analyses are described in Supplemental Tables 1–3.

As LB conditions are used to impose a form of chronic stress to the mother and her litter, we examined whether this early stressor would affect the establishment and maintenance of rhythmicity and output function of the adrenal gland in the offspring. Thus, we analyzed expression of clock genes (*Bmal1, Per2, Cry1 Rev-erbα, Dbp*) and corticosterone (CORT) secretion in neonates, juveniles and adult rats. Results for PND10, PND28 and adult males are displayed in Figs. 3, 5 and 6 respectively, and the corresponding statistical analyses are displayed in Supplemental Tables 1–3. On PND10, Cosine regression analyses showed that all genes were rhythmic in NB pups, but that for both *Bmal1* and *Cry1*, this rhythmicity was abolished in LB pups (Fig. 3). More specifically, *Bmal1* expression was rhythmic in NB pups (F(2, 29) = 3.328, *p* = 0.034 with an R^2^ = 0.263), but not in LB reared pups (F(2, 29) = 0.861, *p* = 0.473 with an R^2^ = 0.084) (Fig. 3A). There was no significant effect of time (F(3, 56) = 2.190, *p* = 0.099), bedding (F(1, 56) = 0.410, *p* = 0.524) or time x bedding interaction. In contrast to *Bmal1*, expression of *Per2* (Fig. 3C) was rhythmic for both NB (F(2, 29) = 6.564, *p* = 0.002 with an R^2^ = 0.413) and LB (F(2, 29) = 9.025, *p* < 0.001 with an R^2^ = 0.492) pups. There was a significant effect of time (F(3, 56) = 15.87, *p* < 0.0001), but no bedding (F(1, 56) = 1.411, *p* = 0.240) or time x bedding interaction. A subset of samples was taken to measure expression levels of *Rev-erbα* (Fig. 3E)*, Dbp* (Fig. 3G) and *Cry1* (Fig. 3I). *Rev-erbα* showed rhythmic expression in both the NB (F(2, 27) = 5.123, *p* = 0.0064 with an R^2^ = 0.372) and LB (F(2,25) = 4.455, *p* = 0.013 with an R^2^ = 0.358) reared pups. There was both a significant effect of time (F (3, 50) = 8.084, *p* < 0.001) and bedding (F (1, 50) = 5.822, *p* = 0.020), but no time x bedding interaction (F(3, 50) = 1.98, *p* = 0.123). LB reared pups displayed an overall increased amplitude of *Rev-erbα* (*p* = 0.0272) compared to NB pups. Similar findings were observed for *Dbp,* where rhythms were observed in both groups (NB: F(2,27) = 6.910, *p* = 0.001 with an R^2^ = 0.444; LB: F(2, 24) = 5.341, *p* = 0.006 with an R^2^ = 0.411). There was an observed effect of time (F (3, 49) = 10.57, *p* < 0.001) and bedding (F (1, 49) = 6.898, *p* = 0.0115). Bonferroni’s test for multiple comparisons yielded a significant group difference at the peak level of expression at CT8 (*p* = 0.0064). An increased amplitude for *Dbp* was close to significant for LB compared to NB pups (*p* = 0.068). Although *Cry1* was found to be rhythmic in NB reared pups (F(2, 26) = 7.056, *p* = 0.0013 with an R^2^ = 0.4585), rhythmicity was not observed in LB reared pups (F(2, 24) = 0.391, *p* = 0.761 with an R^2^ = 0.048). There was a significant effect of time (F (3, 48) = 3.226, *p* = 0.031) and a significant effect of bedding (F (1, 48) = 5.008, *p* = 0.029). Bonferoni’s test for multiple comparisons yielded a significant group difference at CT8 (*p* = 0.0262). Due to the uneven number of males and females in PND10 offspring, we were not able to perform a reasonable statistical analysis of sex differences in this age group.

**Figure 4:**
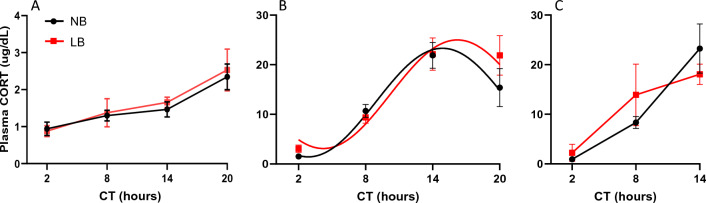
Circadian changes in plasma corticosterone (CORT) concentrations in NB (black) or LB (red) offspring on PND10 (**A**) (n = 8 rats/group/time point), PND28 (**B**) ((n = 11–13 rats/group/time point) and in adult male offspring (**C**) (n = 4–5 rats/group/time point), measured at tissue collection times. CT, circadian time. Values represent the mean +/− SEM.

On PND28, there was a robust circadian rhythmicity for all genes examined in both NB and LB juveniles (pooled male and female data) as assessed by cosine regression analysis (*p* < 0.001) (Fig. 4). ANOVA showed a significant time effect (*p* < 0.01) for all genes examined, but a significant bedding effect only for *Dbp* (F(1, 84) = 6.797, *p* = 0.01, Fig. 4G). For *Dbp*, the interaction between time and bedding was close to significant (F(3, 84) = 2.579, *p* = 0.059) and the LB group displayed an enhanced amplitude compared to the NB group (*p* = 0.0229). At this age, the acrophase for expression of the various genes was similar to that generally found in adult animals^37^. In order to determine whether there were significant sex differences in the expression of circadian genes in PND28 offspring, we performed a 2 way-ANOVA with sex and time as factors, pooling NB and LB offspring as there were no significant bedding effects. A significant sex effect was observed for *Dbp* expression (F(1, 84) = 3.987, *p* = 0.049), but no time x sex interaction. No other genes exhibited significant sex effects, but time x sex interactions were observed for *Per2* (*p* = 0.042, CT20: F > M), *Reverbα* (*p* = 0.027, CT14: F > M) and *Cry-1* (*p* = 0.01,CT14: F > M).

**Figure 5:**
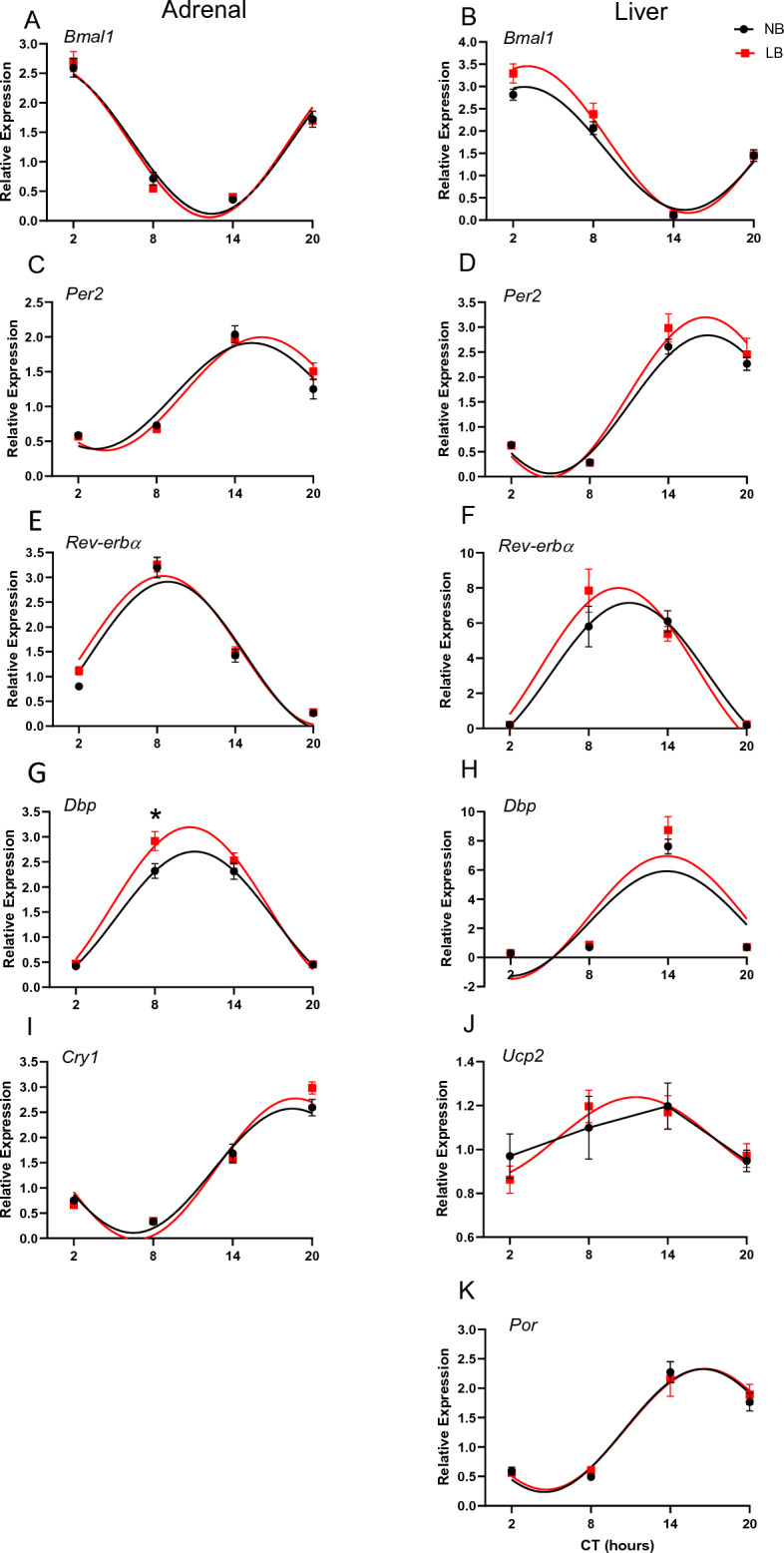
Gene expression of clock and clock-associated genes in the adrenal gland (left, **A**,**C**,**E**,**G**,**I**) and liver (right, **B**,**D**,**F**,**H**,**J**,**K**) of juveniles (PND28) rats raised under NB or LB conditions. Black lines represent data for NB pups while red lines represent data for LB pups. Groups found to display a rhythm according to cosine regression analysis (*p* < 0.05) are represented with curved lines, while those which are not rhythmic are represented by straight lines going directly through the plotted points. For all genes, male and female samples were pooled due to the lack of sex differences and n = 11–13 samples/time point. Statistical significance and cosine regression analyses are described in Supplemental Tables 1–3. CT, circadian time. Values represent the mean +/− SEM. *p < 0.05; **p < 0.01 Tukey HSD post-hoc test.

In adult males, we did not perform cosine regression analysis because we only determined gene expression at 3 time points across the circadian cycle (Fig. 5). However, ANOVA showed a significant time effect for all genes examined (*p* < 0.001) and a significant bedding effect for *Rev-erbα* (F(1, 22) = 15.68, *p* = 0.0007, Fig. 5E) as well as a significant time x bedding interaction (F(2, 22) = 9.18; *p* = 0.0013). At CT2, the expression of *Rev-erbα* was significantly higher in LB compared to NB adult males. Expression of *Cry1* was also significantly higher in LB compared to NB rats at CT14 (*p* < 0.01). There were no other significant bedding differences in other circadian gene expression in adult male offspring.

In summary, significant changes in *Bmal1*, *Rev-erbα, Dbp* and *Cry1* expression observed in neonatal offspring after exposure to LB conditions were mostly transient except for significant bedding effects for *Dbp* in juveniles and *Rev-erbα* and *Cry1* in adults*.* Adverse early conditions did not impact the establishment of adrenal circadian rhythms in juvenile rats.

### Effect of bedding conditions on basal circadian plasma CORT levels

As shown in Fig. 4, basal corticosterone (CORT) concentrations were significantly increasing with time at all ages examined. A two-way ANOVA with bedding and time as between-subjects factors revealed that on PND10 basal CORT concentrations (n = 8 pups/group) were not affected by bedding condition (F (1, 56) = 0.212, *p* = 0.647), as previously demonstrated in our lab^29^. There was a significant effect of time on basal CORT levels (F (3, 56) = 9.193, *p* < 0.001), with concentrations found at CT20 being significantly higher than at CT2. Cosine regression analysis showed a lack of CORT rhythmicity in the LB group (*p* = 0.101), while the NB group exhibit a normal rhythm (*p* = 0.032). On PND28, plasma CORT concentrations displayed significant rhythmicity of (*p* < 0.001) in both bedding groups as well as a significant time effect (F(3, 76) = 24.04; *p* < 0.001) by two way ANOVA. There was no bedding or time x bedding interaction at this age. Finally, in adult males, two way ANOVA revealed a significant time effect (F(2, 22) = 17.73; *p* < 0.001), but no other significant effects. While significant time effects of basal plasma CORT concentrations were observed at all ages, bedding conditions had no significant effect at any age examined.

### Expression of clock genes and metabolism-related genes in the liver is altered by bedding conditions

**Figure 6:**
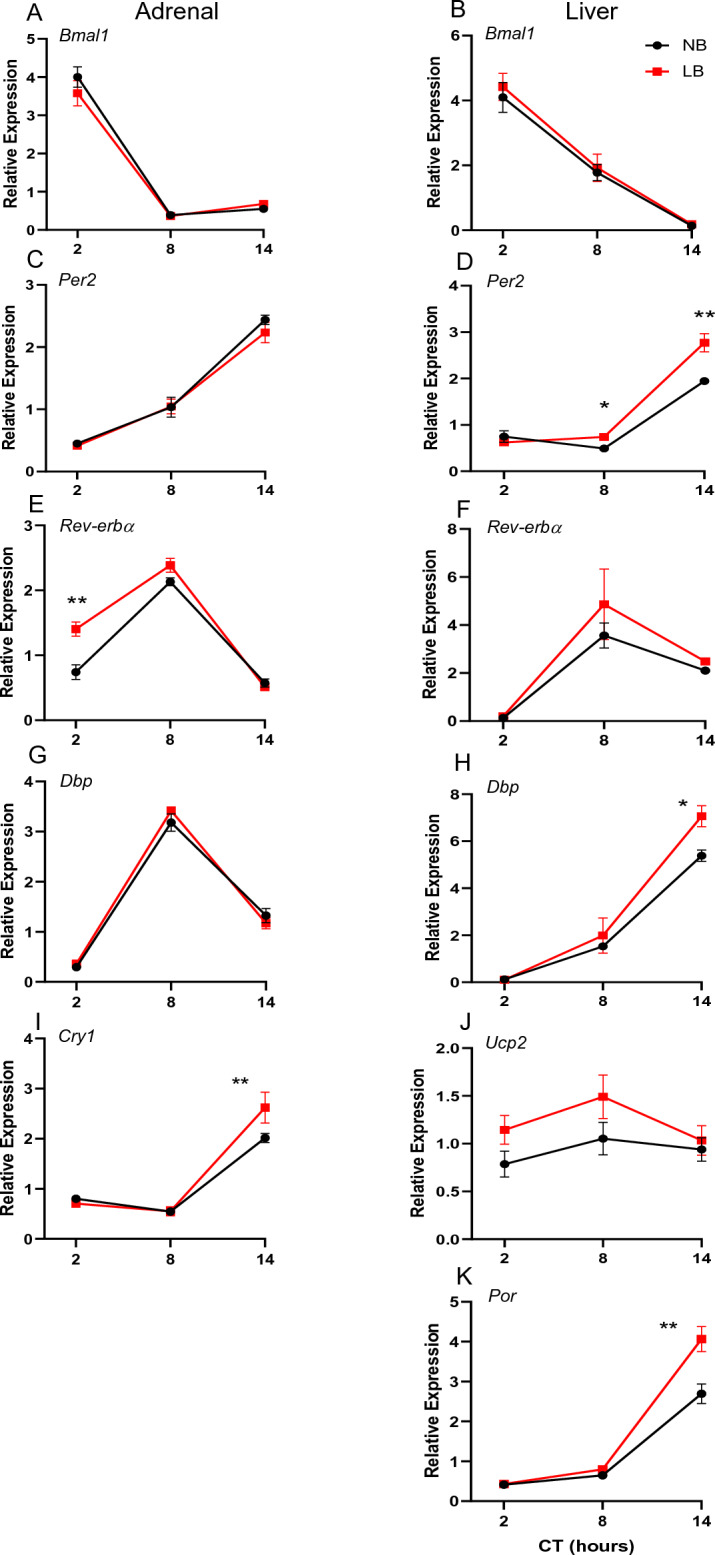
Gene expression of clock and clock-associated genes in the adrenal gland (left, **A**,**C**,**E**,**G**,**I**) and liver (right, **B**,**D**,**F**,**H**,**J**,**L**) of adult male rats raised under NB or LB conditions. Black lines represent data for NB rats while red lines represent data for LB rats. Because we only collected 3 time points, we were unable to perform cosine regression analysis. For all genes, n = 4–5 samples/time point/bedding group. ANOVA analyses are described in Supplemental Table 1. CT, circadian time. Values represent the mean +/− SEM. *p < 0.05; **p < 0.01 Tukey HSD post-hoc test.

Since exposure to limited bedding conditions modified nursing time compared to control conditions, we examined whether this could have consequences for the establishment of clock gene and clock-controlled gene oscillations in the liver. We analyzed the expression of both clock (*Bmal1 and Per2*) and clock related genes that are relevant for metabolic regulation such as *Dbp, Rev-erbα, UCP2,* and *Por.* Results for PND10, PND28 and adult males are displayed in Figs. 3, 5, and 6 respectively and the corresponding statistical analyses are displayed in Supplemental Tables 1–3. On PND10, cosine regression analysis showed that *Bmal1* expression was rhythmic in NB pups (F(2, 26) = 3.14, *p* = 0.043, R^2^ = 0.273), but not in the LB pups (F(2, 29) = 1.20, *p* = 0.32, R^2^ = 0.114). A two-way ANOVA showed a significant effect of time (F(3, 53) = 3.735 *p* = 0.0165) as well as a strong trend for an effect of bedding (F(1, 53) = 3.743, *p* = 0.058) to increase *Bmal1* expression in LB group. There was a strong trend towards rhythmicity for *Per2* for both NB (F(2, 29) = 2.929, *p* = 0.0508, R^2^ = 0.239) and LB pups (F(2, 29) = 2. 90 *p* = 0.0524, R^2^ = 0.237), and while there was a significant effect of time (F (3, 56) = 6.537, *p* < 0.001), no effect of bedding was observed (F(1, 56) = 1.370, *p* = 0.246). A subset of samples was taken to measure expression levels of clock-associated genes *Dbp* and Rev-erb*α*. *Dbp* did not appear to be rhythmic in NB pups (F(2, 18) = 0.33, *p* = 0.802 with an R^2^ = 0.066)), although it was found to be rhythmic in LB pups (F(2, 21) = 3.766, *p* = 0.027 with an R^2^ = 0.361). There was an overall effect of time (F (3, 34) = 4.689, *p* = 0.0076), but no effect of bedding (F (1, 34) = 0.209, *p* = 0.65). There were no rhythms observed for Rev-erb*α* in either the NB (F(2, 15) = 0. 057, *p* = 0. 9812 with an R^2^ = 0.012) or LB (F(2, 21) = 2. 310, *p* = 0.107 with an R^2^ = 0.257) reared pups. A two-way ANOVA showed an overall effect of time (F (3, 34) = 2.93 *p* = 0.048), but no effect of bedding (F(1, 34) = 0.70 *p* = 0.408). Metabolism associated genes in the liver did not exhibit rhythmicity in NB or LB pups, except for *Por*, which showed rhythmicity only in the LB pups (F(2, 29) = 3.315, *p* = 0.034, R^2^ = 0.262). ANOVA analyses showed significant effects of time for *Ucp2* (F(3, 56) = 4.335, *p* = 0.008), and *Por* (F(3, 56) = 4.232, *p* = 0.009), but no significant effect of bedding for either genes.

Similar to the adrenal gene expression, we observed a strong rhythmic expression of all genes examined in the liver of juvenile rats on PND28 (*p* < 0.001), except for *Ucp2* in NB offspring (cosine regression, *p* = 0.33). ANOVA revealed a significant (*p* < 0.001) time effect for all genes examined, but no bedding or time x bedding interactions. To examine sex differences in the expression of circadian genes in PND28 offspring, we performed a 2 way-ANOVA with sex and time as factors, pooling NB and LB offspring as there were no significant bedding effects. None of the liver genes examined at PND28 exhibited a significant sex effect. Significant time effects (*p* < 0.001) were also observed for expression of all genes, except *Ucp2* in adult male rats. Interestingly, in adult male rats, we found significant bedding effects for *Per2* (F(1, 22) = 11.32, *p* = 0.0028), *Dbp* (F(1, 22) = 6.29; *p* = 0.02), *Por* (F(1, 22) = 11.56; *p* = 0.0026) and *Ucp2* (F(1, 22) = 5.24; *p* = 0.032). For all these genes, expression in LB rats was higher at specific time points (CT8 or CT14) compared to NB rats. Overall, circadian expression of clock genes and clock-controlled genes was well established by PND28 in the liver in offspring of both NB and LB mothers, but the effect of bedding condition on several genes was only revealed in adulthood.

### Effects of limited bedding on adult male locomotor activity

In order to determine whether LB conditions could have long term effects on locomotor behavior, we measured wheel running activity in 2 cohorts (cohort 1, n = 12; cohort 2 n = 16) of male rat offspring in various lighting conditions including 12:12LD, DD and LL. Cohort 2 was used as a replication of cohort 1, but data from both cohorts were not merged. In the next sections, we describe the analyses for the second cohort of rats (cohort 2), then compare it to the results obtained in the first cohort (cohort 1, data in Supplementary Tables 3–6) to confirm our findings.

**Figure 7:**
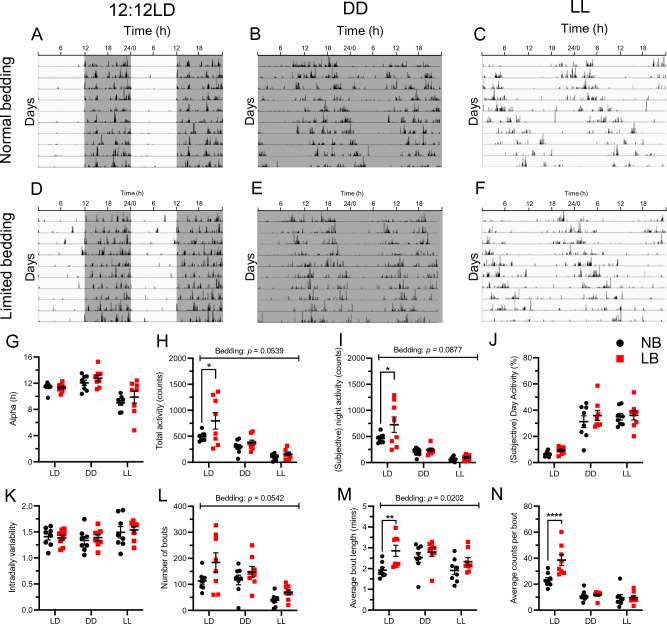
Running wheel activity variables in normal (NB) and limited bedding (LB) adult male offspring from cohort 2. Representative actograms of normal bedding (**A**–**C**) and limited bedding (**D**–**F**) adult offspring under 12:12LD (**A**,**D**), DD (**B**,**E**) and LL (**C**,**F**). Actograms depict locomotor activity; days are vertically stacked one on the other, time (in hours) is shown across the x-axis, and data are double plotted to facilitate visualization. The last 10 days of each condition are shown. The analyzed variables include: (**G**) alpha (h), (**H**) total activity (counts), (**I**) (subjective) night activity (counts), (**J**) (subjective) day activity (%), (**K**) intra-daily variability, (**L**) number of bouts, (**M**) average bout length (mins), and (**N**) average counts per bout. Individual data points represent independent rats and values are mean ± SEM (n = 6 rats/bedding). A two-way ANOVA (factors: bedding × lighting) was used with Sidak’s *post-hoc* tests. If no interaction was found, main effects were explored and presented in the graph. ****p < 0.0001; **p < .01 and *p < .05. Detailed wheel running parameters for cohort 1 and cohort 2 are described in Supplemental Tables 4 and 5, respectively. ANOVA analyses for wheel running variables for cohort 1 and cohort 2 are described in Supplemental Tables 6 and 7, respectively.

Representative actograms for NB and LB offspring (cohort 2) in each lighting condition are shown in Fig. 7A–F and a set of specific parameters are found in Fig. 7G–N. Supplemental Tables 4–7 list all analyzed parameters and two way ANOVA analyses for both cohorts.

LB in rats overall led to a trending increase in total activity (counts) (F(1, 14) = 4.428, *p* = 0.0539) (Fig. 7H), which seemed to be distributed across the (subjective) day (F(1, 14) = 3.724, *p* = 0.0741) (Supplemental Table 7), and (subjective) night (F(1, 14) = 3.371, *p* = 0.0877) (Fig. 7I). Interestingly, post-hoc analyses revealed that the LB-induced increase in total and night activity was driven by differences under LD (LB vs. NB: *p* = 0.0151 and *p* = 0.0151 respectively), whereas the LB-induced increase in (subjective) day activity seemed to be spread out across lighting conditions. No significant interactions were observed in the parameters listed above, and no significant interactions or main effect of bedding were observed in percent (subjective) day activity (F(1, 14) = 1.051, *p* = 0.3226) (Fig. 7J).

Interestingly, it was the microstructure of locomotor activity that was affected, as we found significant bedding × lighting interactions for average counts per bout (F(2, 28) = 6.968, *p* = 0.0010) (Fig. 7N) and average peak rate (F(2, 28) = 3.719, *p* = 0.0369) (Supplemental Table 7), with post-hoc comparisons revealing that both effects were driven by differences in LD (LB vs. NB: *p* < 0.001, *p* = 0.0089 respectively). Further, LB led to greater average bout length (F(1, 14) = 6.862, *p* = 0.0202) (Fig. 7M), and a trend for greater number of bouts (F(1, 14) = 4.416, *p* = 0.0542) (Fig. 7L) and bouts per day (F(1, 14) = 4.434, *p* = 0.0538) (Supplemental Table 7), with no significant interactions. No significant interactions or main effects of bedding were found when considering non-parametric variables (Fig. 7K, Supplemental Table 7), period (h) (Supplemental Table 7) or *alpha* (Fig. 7G). In addition to the DD or LL conditions, we tested a 6 h phase advance as an indirect mean to evaluate the strength of the SCN oscillator and its potential changes after early limited bedding conditions. We found no significant bedding differences for the 6 h phase advance manipulation as shown in Supplemental Figure S1.

In both cohort 1 and 2, we report significant main effects of lighting across rats (irrespective of bedding condition), except in cohort 1 for relative amplitude and intra-daily variability, and in cohort 2 for period (Supplemental Tables 6 and 7). In cohort 1, the only variable with a significant bedding x lighting interaction was *alpha* (F(2, 20) = 13.060, *p* = 0.0002), where post-hocs revealed that LB rats had a significantly longer *alpha* in LL than NB rats (*p* = 0.0001) (Supplemental Table 6). No significant interactions or main effects of bedding were found in other variables for rats in cohort 1 (Supplemental Table 6). Overall, locomotor behavior of male offspring was only modestly impacted by early bedding conditions, showing higher activity in LB offspring compared to NB rats mostly in the LD conditions.

## Discussion

The establishment of circadian rhythmicity is an important process leading to homeostatic regulation adapted to the environment. Maternal circadian rhythms in neuronal activity, hormonal secretion and behavior are critical to the development of circadian activity in the offspring and to the programming of health and disease risk in adulthood^38,39^. In this study, we examined whether disruption in the early environment via limited supply of bedding and nesting material and changes in maternal care might affect the development of circadian rhythmicity of clock genes in the SCN and periphery of the rat offspring. The main findings from this study are that increased fragmentation of maternal behavior and changes in nursing time was associated with significant changes in clock gene expression in the liver and adrenal gland in PND10 offspring and later, some of these remaining altered until adulthood. Adverse conditions did not affect neonatal *Bmal1* gene expression in the SCN, but blunted the amplitude of *Per2* in this brain nucleus. In adulthood, LB conditions affected both macro and microstructure of circadian locomotor behavior in male offspring.

Our early environmental manipulation (LB) imposed during the first 10 days of life increased nursing time during the dark phase and as a result, reduced light/dark phase differences in nursing observed in LB compared to NB mothers on PND5-6. The increased nighttime nursing in LB was also previously documented in PND2-4 mothers^40^. Fragmentation of maternal behavior was observed to be modestly higher in LB mothers during the light phase of the cycle, a behavioral characteristic that can be associated with hypervigilance in LB mothers^41^ and shorter nursing bouts that might reduce milk intake. This was confirmed by the consistent reduction in LB pup weight gain in LB compared to NB neonatal pups, juveniles and even adults in our studies and that of others^29,30,35,40^. Reduced weight gain can be caused by either a reduction in total milk intake and/or hypothermia caused by the LB procedure as previously documented^40^. Both changes in pup core body temperature^40^ and feeding amount and/or timing might significantly alter the circadian expression of clock genes in the offspring as these variables are recognized to affect primarily peripheral oscillators in adults^6^. Lower body weight was maintained in adult LB compared to NB offspring, suggesting that catch-up growth did not occur after exposure to LB conditions.

There is ample evidence that the SCN develops perinatally and that it might be subjected to environmental regulation, mainly through maternal rhythms, feeding and behavior during this maturation period^14,42,43^. Before the rhythmic expression of the canonical clock genes in the SCN, several subsets of genes related to neurodevelopment and cell-to-cell signaling have been identified to display rhythmic expression in the SCN and be influenced by maternal rhythmic signals^14^. However, it is only during the postnatal period that rhythms in *Bmal1* and *Per1* genes have been observed in vivo in the rat SCN^42,44^. Accordingly, we observed a robust rhythmicity in *Bmal1* and *Per2* expression in the SCN in both NB and LB offspring. Despite maternal behavioral disruptions provoked by the LB conditions (nursing, fragmentation) and a significant phase shift in nursing time, mothers maintained a robust overall diurnal rhythm in maternal care, which might have contributed to the rhythmicity of *Bmal1* and *Per2* in the offspring SCN. Interestingly, early LB conditions significantly reduced the amplitude of *Per2* expression, in particular at CT8 and CT14, a time that was associated with greater maternal behavior fragmentation in LB mothers compared to NB mothers (Fig. 1B). Whether this reduced expression of *Per2* has consequences on the downstream clock functioning still needs to be determined. The molecular clock of the neonatal SCN is quite resistant to perturbations as demonstrated by only marginal changes in SCN rhythms of *Per1* and *Per2* expression in blind pups reared by a foster mother entrained on the opposite LD cycle as the original mother from birth on^45^. In contrast to our LB conditions, only major disruptions of the pup’s environment such as complete maternal deprivation during the light phase can reverse the phase of *Per1* and *Per2* expression in blind pups on PND6^43^. Interestingly, earlier studies have suggested that the resilience of the neonatal SCN *Bmal1* rhythm can be challenged by intense stress and increased glucocorticoid secretion early after birth as shifts in *Bmal1*, but not *Per1* or *Per2* expression were observed in the offspring SCN^46^. In our study, exposure to LB conditions did not raise baseline corticosterone concentrations in pups, but it shifted the phase of *Bmal1* expression in the SCN to an earlier peak (CT8) than that observed in 4 day-old Wistar neonates (CT20)^46^ or adults where *Bmal1* is in antiphase with *Per2*. In our study, the peak of *Per2* expression (CT8) was similar to that reported earlier in 4 day-old neonates^46^ and in adults, suggesting that the phase of *Bmal1* rhythm in the offspring SCN is more labile and sensitive to shifts in maternal nursing time (LB group) or other environmental factors than that of *Per2*.In view of the delayed maturation of peripheral oscillators compared to the SCN^44,47^, we sought to determine whether peripheral oscillators in the adrenal gland and the liver would be more sensitive than the SCN to environmental conditions and maternal behavioral changes induced by the LB conditions.

In the adrenal gland, NB pups displayed significant rhythmicity in *Bmal1, Per2, Rev-erbα, Cry1* and *Dbp* gene expression and only *Bmal1* and *Cry1* did not meet rhythmicity criterion of the Cosine regression analysis in LB pups. Expression of *Bmal1* in NB showed a peak expression at CT14, in between the values reported previously for PND6 (ZT12) and PND14 (ZT20)^48^, suggesting that the phase of *Bmal1* in the adrenal is slowly shifting to a more adult-like pattern reached around PND14 for this gene^48^. The *Bmal1* rhythm was abolished in LB offspring, but this effect did not modify the low amplitude circadian production of corticosterone found at this age. This is consistent with previous studies showing that the adrenal deletion of *Bmal1* did not modify the endogenous circadian production of free corticosterone in the adult mouse^49^. Components of the negative limb of the molecular clock were differentially affected by the LB procedure. While the rhythm of *Cry1* was eliminated, that of *Per2* was not modified, exhibiting peak and nadir gene expression that are observed by PND14-16^48^. Several studies have used the mPER2:LUC mouse to investigate the regulation of adult adrenal rhythms under stress conditions. While acute stress exposure is able to phase shift the adrenal clock (*Per2*) rhythm^50^, chronic stress increased the amplitude of the adrenal Per2:LUC rhythm in vitro^51^, more so in females than males. In our studies with pooled male and female pups, we observed only a trend towards a higher amplitude in *Per2* expression in the LB condition. Interestingly, adrenal responsiveness to ACTH has been found to be inversely proportional to the amplitude of the adrenal PER::LUC rhythms in vitro^51^. When transposed to our in vivo data, it is notable that a good amplitude of the adrenal *Per2* rhythm is already observed at a time of low adrenal responsiveness to ACTH during the stress hyporesponsive period in PND10 neonates^52^. This might indicate that the well-established circadian rhythm in *Per2* might contribute to maintain developmental adrenal hyporesponsiveness and thus, serve an adaptive function during development.

Exposure to LB conditions during the time of circadian maturation (PND1-10) significantly increased the amplitude of the rhythm for *Rev-erbα, Dbp and Cry1* in the adrenal gland even though the amplitude of the rhythm in NB controls was robust. For *Rev-erbα*, a peak was observed at CT8, in-between peaks documented for PND6 (ZT18) and PND14 (ZT6), but consistent with peaks observed in juvenile (PND28) rats in the current study. For *Cry1*, a peak at CT14 was similar to that found in PND14 pups (ZT14)^48^, but differed from peak amplitude found at CT20 in juveniles (PND28). The significance of an increased amplitude of rhythmic genes by the chronic stress of LB in neonates is unclear. An increased amplitude is generally associated with higher developmental stage and might therefore indicate an accelerated maturation of some of the molecular clock components in the adrenal gland. Alternatively, the increased amplitude of *Cry1* and *Rev-erbα* might participate, together with *Per2*, in maintaining adrenal hyporesponsiveness in challenging situations such as the LB conditions. Future experiments will be necessary to distinguish between these alternatives.

Interestingly, rhythmicity of all adrenal genes examined was well established for both bedding groups by PND28 with peak and nadir values similar to the adult rhythms and a robust circadian rhythm of CORT secretion. In agreement with previous studies^48^, the phase of *Per2* and *Cry1* expression in juveniles was similar to that of CORT, but we did not find significant bedding effects on expression of these genes or CORT secretion at that age. However, *Cry1* expression was increased in both neonates and adult LB offspring, showing a potential long lasting disruption of the adrenal clock machinery. Although we did not observe major changes in *Cry1* rhythms under constant darkness conditions, it is possible that the consequences of early disruption in core clock gene might be best evidenced under challenging lighting conditions such as LL^53^ or “jet lag” type of changes. Nuclear receptor *Rev-erbα* also exhibited a significantly higher rhythm amplitude in neonates and higher expression at CT2 in adult LB offspring, while *Dbp* expression across the circadian cycle was higher in LB neonates and juveniles, but changes disappeared in adulthood.

Rhythms in clock and clock-controlled genes in the liver were less robust in NB controls than in the adrenal gland on PND10, in agreement with previous reports^54^ demonstrating the emergence of robust circadian activity for all main clock genes in the rat liver by PND30 only. In the liver, only *Bmal1* (and *Per2*, *p* = 0.0508) displayed circadian rhythmicity with a nadir at CT14 in NB pups similarly to studies in mice showing a significant rhythm of *Bmal1* on PND15 while other clock genes or clock output genes are not rhythmic at this age^54^. Interestingly, circadian rhythmicity in liver *Bmal1* was eliminated by LB conditions that lead to a reduction in body weight gain and potential changes in milk intake and/or thermoregulatory processes. In contrast, in the case of neonatal overfeeding (via small litters) or maternal obesity (via high fat diet feeding during pregnancy and lactation) in mice, *Bmal1* expression remained significantly rhythmic^55^ and even displayed an increased amplitude in offspring of obese mothers on PND17^56^. Taken together, these data suggest that maternal feeding prenatally and postnatally can significantly alter the developmental rhythm of *Bmal1* expression and that of other genes such as *Rev-erba* for instance^18^ and that changes in neonatal body weight gain can act as a proxy of the effects of maternal nutrition on emerging rhythms in the liver. This might remains true after weaning as the observed changes in *Dbp*, *Per2* and *Por* gene expression in adulthood are associated with a consistent reduction in body weight of LB offspring.

In neonates, *Dbp* and the clock-controlled gene *Por* exhibited circadian rhythmicity after LB conditions, while none of the core clock genes, except *Bmal1* and *Per2*, or other genes investigated, were modified after LB conditions. Rhythms of *Dbp* expression are generally used to assess the amplitude of circadian rhythms because this gene exhibits robust and high amplitude rhythms in the liver^57^ and might regulate key enzymes in cholesterol metabolism. The emergence of rhythmicity in this gene after exposure to LB conditions might indicate that some aspects of cholesterol metabolism are altered by the LB conditions in neonates. This is further emphasized by the significant *Por* rhythm in LB neonates since POR (P450 oxydo-reductase) is a key enzyme in P450 function and cholesterol metabolism. In adult rats, *Por* circadian rhythm is significantly affected by the time of feeding^58^, an observation that might be relevant to differences in nursing time (and likely milk intake) we found between NB and LB mothers. Increased *Por* expression in adult LB offspring might also signal changes in food intake patterns compared to NB rats that need to be further investigated.

There are some limitations to our current study. First, we determined gene expression rhythms based on four time points in the 24 h cycle and a larger number of sampling times might have provided a more sensitive assessment of rhythmicity in gene expression. Second, based on former literature showing the lack of sex effects on developmental circadian rhythms, we pooled male and female PND10 and PND28 offspring in our samples. A separate analysis of sex effects in juveniles, pooling both bedding conditions revealed that sex by time interactions existed for some of the genes in the adrenal gland (*Per2, Rev-erbα* *and Cry1)*, but not the liver. Recent studies in adult rodents have determined that sex differences might exist in the liver as some hepatic clock genes (*Per 1, Per2, Cry2*, but not *Rev-erbα*) exhibit an advanced acrophase in females and lower amplitude in male mice^18^. Female rats are also less vulnerable to diet-induced circadian disruption of hepatic metabolism compared to males^59^. Since we tested only adult male rats in the current study, we are not able to confirm these previous results, but further studies in adult NB and LB offspring are ongoing to investigate how the emergence of sex differences on circadian rhythms might be impacted by early life adversity.

In summary, our data demonstrate that being raised under early adverse environmental conditions has a significant effect on the establishment of yet immature peripheral rhythms in the adrenal gland and the liver, but only affected *Per2* rhythm amplitude in the SCN. Changes in nursing time and fragmentation of maternal behavior, together with the difficulty to maintain adequate temperature in pups during the LB exposure might provide important signals to the maturing peripheral oscillators, in particular to adapt corticosterone secretion and liver metabolism of cholesterol. Some of the changes in gene expression observed during neonatal life were maintained in juveniles and/or adult LB offspring. In adult offspring, alterations in the microstructure of locomotor behavior might derive from changes in the circadian pattern of clock gene expression in the brain, beyond those documented here in the neonatal SCN. Future studies should examine the precise contribution of environmental factors to the immediate and long-term development of central and peripheral clocks after early adversity.

### Supplementary Information

Supplementary Tables.

Supplementary Figure S1.

## Data Availability

The datasets generated and analyzed during the current study are available from the corresponding author upon reasonable request.
