# Supplementary material for: Peripheral clock gene oscillations are perturbed in neonatal and adult rat offspring raised under adverse limited bedding conditions
Source: Sci Rep. 2023 Dec 21;13:22886. doi: 10.1038/s41598-023-47968-y (PMC10739797; doi:10.1038/s41598-023-47968-y)
Supplement: Supplementary file 1 — Supplementary Tables. [file 41598_2023_47968_MOESM1_ESM.pdf]

Supplemental Table 1. Summary of two-way ANOVAs to explore treatment x lighting interactions in circadian clock and clock controlled genes in the liver and adrenal gland of neonates, juvenile and adult rats.

		Time x Bedding		Main Effect of Bedding		Main Effect of Time	
		Liver	Adrenal	Liver	Adrenal	Liver	Adrenal
	Age	*p-value*	*p-value*	*p-value*	*p-value*	*p-value*	*p-value*
Bmal1	PND10	0.341	0.263	***0.058***	0.524	**0.017**	0.099
	PND28	0.3	0.643	***0.053***	0.871	**0.001**	**0.001**
	Adult	0.9	0.333	0.515	0.513	**0.001**	**0.001**
Per2	PND10	0.648	0.582	0.247	0.24	**0.001**	**0.001**
	PND28	0.618	0.219	0.266	0.668	**0.001**	**0.001**
	Adult	**0.001**	0.572	**0.003**	0.389	**0.001**	**0.001**
Rev-erbα	PND10	0.602	0.13	0.408	**0.02**	**0.048**	**0.001**
	PND28	0.161	0.52	0.441	0.151	**0.001**	**0.001**
	Adult	0.537	**0.001**	0.200	**0.007**	**0.001**	**0.001**
Dbp	PND10	0.456	0.12	0.651	**0.012**	**0.008**	**0.001**
	PND28	0.435	***0.059***	0.231	**0.011**	**0.001**	**0.001**
	Adult	***0.051***	0.212	**0.02**	0.57	**0.001**	**0.001**
Cry1	PND10	-	0.121	-	**0.03**	-	**0.031**
	PND28	-	***0.066***	-	0.503	-	**0.001**
	Adult	-	***0.059***	-	0.186	-	**0.001**
Por	PND10	0.339	-	0.761	-	**0.009**	-
	PND28	0.836	-	0.85	-	**0.001**	-
	Adult	**0.002**	-	**0.003**	-	**0.001**	-
Ucp2	PND10	0.506	-	0.309	-	**0.008**	-
	PND28	0.69	-	0.955	-	**0.004**	-
	Adult	0.532	-	**0.032**	-	0.14	-
Plasma CORT	PND10	-	0.971	-	0.647	-	**0.001**
	PND28	-	0.461	-	0.334	-	**0.001**
	Adult	-	0.295	-	0.838	-	**0.001**

Notes: Results from two-way ANOVAs for gene expression in the liver and adrenal gland, highlighting the treatment x circadian time interactions and main effects of treatment (NB or LB) and circadian time (CT) at various ages tested. P-values in **bold** are $p<0.05$, and ***bold/italics*** are $p<0.1$.

Supplemental Table 2. Cosine regression analysis of circadian liver and adrenal gene expression in neonates and juvenile rats

		Liver				Adrenal			
		NB		LB		NB		LB	
	Age	F	*p-value*	F	*p-value*	F	*p-value*	F	*p-value*
Bmal1	PND10	3.52	**0.028**	1.202	0.327	3.329	**0.034**	0.861	0.473
	PND28	82.08	**0.001**	60.5	**0.001**	61.3	**0.001**	81.17	**0.001**
Per2	PND10	2.931	***0.051***	2.899	***0.052***	6.56	**0.002**	9.03	**0.001**
	PND28	92.95	**0.001**	33.4	**0.001**	32	**0.001**	69.2	**0.001**
Rev-erbα	PND10	0.114	0.950	2.311	0.107	5.53	**0.004**	5.204	**0.005**
	PND28	28.6	**0.001**	32.66	**0.001**	63.09	**0.001**	92.06	**0.001**
Dbp	PND10	0.659	*0.058*	0.332	0.802	7.45	**0.001**	6.502	**0.002**
	PND28	27.66	**0.001**	21.44	**0.001**	97.4	**0.001**	104.6	**0.001**
Cry1	PND10	--	--	--	--	7.88	**0.001**	0.47	0.705
	PND28	--	--	--	--	57.8	**0.001**	98	**0.001**
Por	PND10	1.607	0.209	3.315	**0.034**	--	--	--	--
	PND28	41.23	**0.001**	21.75	**0.001**	--	--	--	--
Ucp2	PND10	1.18	0.336	1.83	0.164	--	--	--	--
	PND28	1.156	0.33	5.21	**0.001**	--	--	--	--

Notes: Number of points used in cosine regression analysis is n=32 (PND10), n=46 (PND28). Cosine regression analysis was not performed in adult rats. NB, Normal bedding; LB, limited bedding. P-values in **bold** are p<0.05, and ***bold/italics*** are p<0.1.

Supplemental Table 3. Parameters of significant cosine regression analysis of circadian liver and adrenal gene expression in neonates and juvenile rats

		Liver						Adrenal					
		NB			LB			NB			LB		
	Age	Baseline	Amplitude	Phase shift	Baseline	Amplitude	Phase shift	Baseline	Amplitude	Phase shift	Baseline	Amplitude	Phase Shift
Bmal1	PND10	0.948	0.177	22.45	---	---	---	0.977	0.19	16.00	---	---	---
	PND28	1.609	1.381	2.841	1.808	1.648	3.087	1.348	1.229	0.407	1.344	1.285	0.224
Per2	PND10	1.024	0.331	12.32	1.137	0.249	10.57	0.958	0.293	14.28	1.042	0.394	14.03
	PND28	1.450	1.385	17.01	1.594	1.603	16.84	1.152	0.761	15.30	1.183	0.813	16.05
Rev-erbα	PND10	---	---	---	---	---	---	0.938	0.398	5.91	1.485	1.097	6.56
	PND28	3.076	4.073	11.09	3.390	4.607	10.28	1.420	1.490	8.841	1.525	1.502	8.472
Dbp	PND10	1.231	0.276	13.14	1.214	0.539	8.97	0.94	0.451	6.09	1.407	0.89	8.0
	PND28	2.323	3.596	13.92	2.739	4.218	13.93	1.376	1.330	11.03	1.588	1.604	10.66
Cry1	PND10	---	---	---	---	---	---	0.933	0.286	14.28	---	---	---
	PND28	---	---	---	---	---	---	1.340	1.232	18.49	1.378	1.396	18.73
Por	PND10	---	---	---	1.083	0.316	10.91	---	---	---	---	---	---
	PND28	1.281	1.046	16.47	1.304	1.027	16.61	---	---	---	---	---	---
Ucp2	PND10	---	---	---	---	---	---	---	---	---	---	---	---
	PND28	---	---	---	1.049	0.189	11.58	---	---	---	---	---	---

Notes: Parameters for cosine regression analysis were only shown for significant values of cosine as per 2-way ANOVA (Supplemental Table 2). Cosine regression analysis was not performed for adult rats.

Supplementary Table 4. Summary of wheel running parameters from limited bedding and normal bedding male rats from cohort 1

	Cohort 1					
	12:12LD		DD		LL	
	NB (n=6)	LB (n=6)	NB (n=6)	LB (n=6)	NB (n=6)	LB (n=6)
**Period (h)**	24.06 ± 0.055	24.0 ± 0.0745	24.36 ± 0.051	24.42 ± 0.148	25.58 ± 0.340	25.67 ± 0.172
**Alpha (h)**	11.14 ± 0.278	11.13 ± 0.222	11.2 ± 0.277	11.07 ± 0.681	7.546 ± 0.766	11.82 ± 0.539
**(Subjective) Day Activity (counts)**	29.91 ± 11.06	27.83 ± 9.058	39.68 ± 17.91	25.73 ± 4.342	8.433 ± 3.548	8.535 ± 1.618
**(Subjective) Night Activity (counts)**	480.3 ± 214.3	498.6 ± 153.1	233 ± 35.66	234 ± 47.55	67.10 ± 26.01	57.90 ± 15.88
**Total Activity (counts)**	510.2 ± 213.9	526.4 ± 160	272.7 ± 39.27	259.7 ± 48.55	75.53 ± 28.12	66.43 ± 17.26
**(Subjective) Day Activity (%)**	8.509 ± 2.176	6.034 ± 0.922	11.42 ± 3.514	11.27 ± 2.864	13.10 ± 1.923	14.79 ± 2.653
**Relative Amplitude**	0.9373 ± 0.026	0.964 ± 0.007	0.930 ± 0.016	0.918 ± 0.019	0.826 ± 0.079	0.902 ± 0.024
**Intradaily Variability**	1.285 ± 0.125	1.347 ± 0.096	1.289 ± 0.036	1.406 ± 0.090	1.255 ± 0.130	1.596 ± 0.135
**Interdaily Stability**	0.521 ± 0.082	0.578 ± 0.035	N/A	N/A	N/A	N/A
**Onset Variability**	0.785 ± 0.335	0.675 ± 0.176	N/A	N/A	N/A	N/A
**Number of Bouts**	44.17 ± 3.321	46 ± 3.983	43.33 ± 3.509	47.50 ± 1.839	21.33 ± 5.175	22.33 ± 4.602
**Average Bout Length (minutes)**	62.41 ± 13.08	55.12 ± 15.17	46.59 ± 5.955	38.06 ± 4.908	19.84 ± 7.029	17.86 ± 4.726
**Average Counts per Bout**	94.78 ± 48.67	90.08 ± 33.31	34.18 ± 5.120	31.15 ± 7.235	14.96 ± 4.402	12.35 ± 2.698
**Average Peak Rate**	9.497 ± 2.311	10.58 ± 2.166	5.702 ± 0.337	5.722 ± 0.593	4.4 ± 0.265	4.177 ± 0.257

Notes: Values represent mean ± SEM. N/A denotes parameters that were not assessed in constant conditions. 12:12LD, 12 h of light, 12 h of dark; DD, constant dark; LL, constant light; NB, normal bedding; LB, limited bedding.

Supplementary Table 5. Summary of wheel running parameters from limited bedding and normal bedding male rats from cohort 2.

	Cohort 2					
	12:12LD		DD		LL	
	NB (n=8)	LB (n=8)	NB (n=8)	LB (n=8)	NB (n=8)	LB (n=8)
**Period (h)**	24.0 ± 0.00	24.0 ± 0.00	23.6 ± 0.02	23.7 ± 0.02	21.17 ± 3.240	24.38 ± 1.346
**Alpha (h)**	11.43 ± 0.246	11.34 ± 0.227	13.7 ± 0.36	14.9 ± 0.28	9.015 ± 0.378	9.888 ± 0.925
**(Subjective) Day Activity (counts)**	31.15 ± 4.680	58.39 ± 12.46	2499 ± 306	4123 ± 321	34.56 ± 5.472	58.96 ± 17.88
**(Subjective) Night Activity (counts)**	459.9 ± 32.85	636.9 ± 147	18276 ± 1563	16265 ± 1301	66.21 ± 15.51	95.58 ± 17.80
**Total Activity (counts)**	491.1 ± 34.15	695.3 ± 154.3	20564 ± 1486	20180 ± 1263	100.7 ± 19.14	156.4 ± 31.97
**(Subjective) Day Activity (%)**	6.578 ± 0.954	9.028 ± 1.083	13.25 ± 2.09	21.18 ± 1.98	34.91 ± 2.428	36.02 ± 3.488
**Relative Amplitude**	0.939 ± 0.013	0.925 ± 0.009	0.968 ± 0.012	0.970 ± 0.010	0.831 ± 0.092	0.782 ± 0.090
**Intradaily Variability**	1.409 ± 0.063	1.387 ± 0.054	0.812 ± 0.107	1.08 ± 0.083	1.493 ± 0.108	1.537 ± 0.066
**Interdaily Stability**	0.502 ± 0.028	0.404 ± 0.042	N/A	N/A	N/A	N/A
**Onset Variability**	0.666 ± 0.219	0.697 ± 0.167	N/A	N/A	N/A	N/A
**Number of Bouts**	113.5 ± 12.40	184 ± 37.08	125.5 ± 11.03	128.5 ± 9.05	39.25 ± 8.478	69.25 ± 9.924
**Average Bout Length (minutes)**	1.928 ± 0.128	2.854 ± 0.269	52.9 ± 6.14	49.4 ± 4.41	1.905 ± 0.199	2.331 ± 0.191
**Average Counts per Bout**	23.20 ± 1.838	38.56 ± 4.122	2509 ± 308	2290 ± 253	9.649 ± 2.294	9.636 ± 1.526
**Average Peak Rate**	14.83 ± 0.431	18.58 ± 0.998	72.46 ± 2.34	74.11 ± 1.61	6.713 ± 1.516	5.975 ± 0.644

Notes: Values represent mean ± SEM. N/A denotes parameters that were not assessed in constant conditions. 12:12LD, 12 h of light, 12 h of dark; DD, constant dark; LL, constant light; NB, normal bedding; LB, limited bedding.

Supplementary Table 6. Summary of two-way ANOVAs to explore treatment x lighting interactions in circadian locomotor variables from cohort 1.

	Cohort 1					
	Treatment x Lighting		Main Effect of Lighting		Main Effect of Treatment	
	F	*p-value*	F	*p-value*	F	*p-value*
**Period (h)**	0.112	0.894	57.13	**0.001**	0.029	0.868
**Alpha (h)**	13.060	**0.0002**	5.845	**0.010**	9.802	**0.011**
**(Subjective) Day Activity (counts)**	0.682	0.517	8.082	**0.003**	0.216	0.652
**(Subjective) Night Activity (counts)**	0.008	0.992	7.279	**0.004**	0.001	0.970
**Total Activity (counts)**	0.010	0.990	7.706	**0.003**	0.000	0.983
**(Subjective) Day Activity (%)**	0.062	0.940	14.53	**0.0001**	0.111	0.746
**Relative Amplitude**	0.821	0.454	3.442	***0.052***	0.765	0.402
**Intradaily Variability**	1.082	0.358	0.628	0.544	3.165	0.106
**Number of Bouts**	0.088	0.916	23.92	**0.0001**	0.556	0.473
**Average Bout Length (minutes)**	0.074	0.929	9.797	**0.001**	0.518	0.488
**Average Counts per Bout**	0.001	0.999	5.447	**0.0129**	0.032	0.861
**Average Peak Rate**	0.138	0.872	10.24	**0.0009**	0.071	0.795
**Bouts per day**	0.133	0.876	35.59	**0.0001**	0.667	0.433

Notes: Results from two-way ANOVAs for circadian locomotor variables, highlighting the treatment x lighting interactions and main effects of treatment and lighting. ANOVA factors include treatment (limited bedding, normal bedding), lighting (12 h of light, 12 h of dark (12:12LD), constant dark (DD) and constant light (LL)). P-values in **bold** are $p<0.05$, and ***bold/italics*** are $p<0.1$.

Supplementary Table 7. Summary of two-way ANOVAs to explore treatment x lighting interactions in circadian locomotor variables from cohort 2.

	Cohort 2					
	Treatment x Lighting		Main Effect of Lighting		Main Effect of Treatment	
	F	*p-value*	F	*p-value*	F	*p-value*
**Period (h)**	0.822	0.449	0.917	0.412	0.867	0.368
**Alpha (h)**	0.803	0.458	26.960	**0.001**	0.910	0.356
**(Subjective) Day Activity (counts)**	0.136	0.874	11.060	**0.003**	3.724	***0.074***
**(Subjective) Night Activity (counts)**	2.515	***0.099***	39.580	**0.001**	3.371	***0.088***
**Total Activity (counts)**	2.191	0.131	31.290	**0.001**	4.428	***0.054***
**(Subjective) Day Activity (%)**	0.216	0.807	62.160	**0.001**	1.051	0.323
**Relative Amplitude**	0.074	0.929	3.460	**0.045**	0.500	0.491
**Intradaily Variability**	0.234	0.793	3.399	**0.048**	0.108	0.747
**Number of Bouts**	0.909	0.414	17.430	**0.001**	4.416	***0.054***
**Average Bout Length (minutes)**	1.761	0.190	4.365	**0.022**	6.862	**0.020**
**Average Counts per Bout**	6.968	**0.004**	52.250	**0.001**	9.299	**0.009**
**Average Peak Rate**	3.719	**0.037**	94.540	**0.001**	2.749	0.119
**Bouts per day**	0.190	0.828	16.860	**0.001**	4.434	***0.054***

Notes: Results from two-way ANOVAs for circadian locomotor variables, highlighting the treatment x lighting interactions and main effects of treatment and lighting. ANOVA factors include treatment (limited bedding, normal bedding), lighting (12 h of light, 12 h of dark (12:12LD), constant dark (DD) and constant light (LL)). P-values in **bold** are $p<0.05$, and ***bold/italics*** are $p<0.1$.
