# Supplementary material for: Peripheral clock gene oscillations are perturbed in neonatal and adult rat offspring raised under adverse limited bedding conditions
Source: Sci Rep. 2023 Dec 21;13:22886. doi: 10.1038/s41598-023-47968-y (PMC10739797; doi:10.1038/s41598-023-47968-y)
Supplement: Supplementary file 2 — Supplementary Figure S1. [file 41598_2023_47968_MOESM2_ESM.pdf]

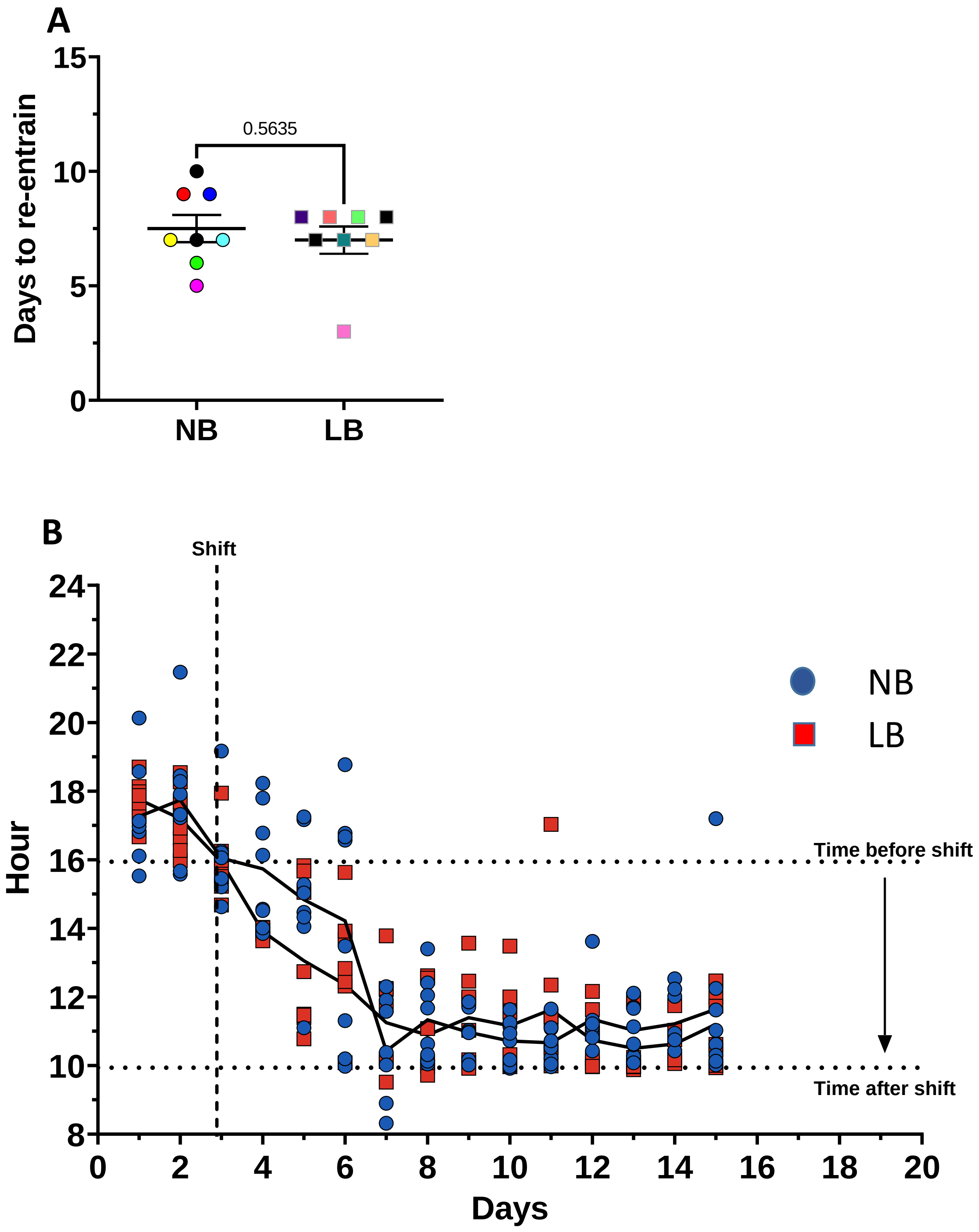

Supplemental Figure 1: (A): Average days required to re-entrain activity levels at the onset of daily activity in NB and LB adult male rats after a 6hr phase advance. Values are mean+/- SEM, n=8 rats/bedding group (B): Time course of the shift in wheel activity after a 6hr phase advance in NB (circles) and LB (square) adult male rats. Individual values are plotted as a function of days. Three days before the 14 days of phase shift are indicated. NB, normal bedding; LB, limited bedding.
